# Cellular and Molecular Mechanisms of 3,3′-Diindolylmethane in Gastrointestinal Cancer

**DOI:** 10.3390/ijms17071155

**Published:** 2016-07-19

**Authors:** Soo Mi Kim

**Affiliations:** Department of Physiology, Chonbuk National University Medical School, Jeonju 561-180, Korea; soomikim@jbnu.ac.kr; Tel.: +82-63-270-3077; Fax: +82-63-274-9892

**Keywords:** 3,3′-diindolylmethane, indolyl-3-carbinol, gastrointestinal cancers, prevention, therapy, apoptosis

## Abstract

Studies in humans have shown that 3,3′-diindolylmethane (DIM), which is found in cruciferous vegetables, such as cabbage and broccoli, is effective in the attenuation of gastrointestinal cancers. This review presents the latest findings on the use, targets, and modes of action of DIM for the treatment of human gastrointestinal cancers. DIM acts upon several cellular and molecular processes in gastrointestinal cancer cells, including apoptosis, autophagy, invasion, cell cycle regulation, metastasis, angiogenesis, and endoplasmic reticulum (ER) stress. In addition, DIM increases the efficacy of other drugs or therapeutic chemicals when used in combinatorial treatment for gastrointestinal cancer. The studies to date offer strong evidence to support the use of DIM as an anticancer and therapeutic agent for gastrointestinal cancer. Therefore, this review provides a comprehensive understanding of the preventive and therapeutic properties of DIM in addition to its different perspective on the safety of DIM in clinical applications for the treatment of gastrointestinal cancers.

## 1. Introduction

Cancer is a leading cause of death, resulting in millions of deaths per year worldwide [1]. Although medical research has significantly advanced in recent years, attempts to understand cancer using different approaches have been negligible [2]. Earlier and more accurate diagnosis, lifestyle, and food habits have slightly improved the overall survival of cancer patients; however, cancers, such as pancreatic or gastrointestinal cancers, which are usually detected at a late stage, continue to result in high mortality rates [3]. Although the incidence of gastrointestinal cancers has declined with improvements in lifestyle and diet, the disease is generally diagnosed at an advanced stage, leading to a poor prognosis and limited treatment approaches [4,5,6,7,8]. Surgical resection is frequently the only choice and confers an overall survival (OS) of <1 year [6,7,8,9,10,11,12,13]. To enhance the survival rate, many studies have explored the molecular mechanisms underlying gastrointestinal cancer or investigated biomarkers of cancer prognosis and responsiveness to treatment. However, to date, the results have been disappointing because of genomic instability, changes in the microenvironment caused by aberrant signaling by multiple pathways, or the speed at which cancer cells acquire resistance to chemotherapeutic agents. Therefore, novel strategies to improve current therapeutic treatments must be explored.

**Figure 1:**
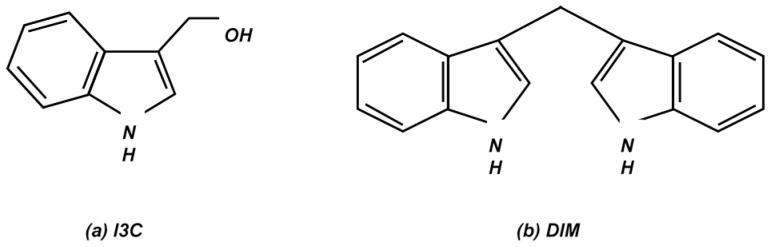
Molecular structure of (**a**) indole-3-carbinol (I3C) and (**b**) 3,3′-diindolylmethane (DIM). Indole-3-carbinol is promptly transformed into the dimeric bioactive product, 3,3′-diindolylmethane in the acidic environment of the stomach following consumption of cruciferous vegetables. Abbreviations: I3C, indole-3-carbinol; DIM, 3,3′-diindolylmethane.

Over the past few decades, many studies have examined the effects of natural compounds such as those found in cruciferous vegetables on various cancers, including gastrointestinal cancer. Cruciferous vegetables have been known to possess anticancer properties since ancient times. The Roman statesman Cato the Elder stated: “If a cancerous ulcer appears on the breasts, apply a crushed cabbage leaf and it will make it well” [2,14]. It has been >25 years since the inverse relationship between nutrition and cancer progression was established [15,16,17,18]. Research conducted in the US has shown that a regular intake of cruciferous vegetables is associated with a decreased risk of colorectal cancer [19]. In addition, epidemiologic investigations in the US, Sweden, and China have found that cruciferous vegetable intake is considerably lower in women diagnosed with breast cancer than in the control group [20]. Vegetable and fruit juices contain large amounts of antioxidants and plant enzymes [21]. Natural fruits and vegetables contain antioxidants, fatty acids, amino acids, and related compounds such as flavonoids, resveratrol, and alkaloids [21]. Exhaustive research on the effects of natural compounds on multiple signaling pathways has revealed the potential of these compounds. The results of these studies support further research to test the chemopreventive qualities of these compounds and find other biochemical targets. Because the study of the therapeutic mechanisms through which these natural compounds act is still in its preliminary state, natural compounds are rapidly being tested for their cancer-curing and preventive properties. Among various natural compounds, studies have shown 3,3′-diindolylmethane (DIM) could be a promising candidate as a therapeutic substance for the treatment of numerous cancers [22,23]. Currently used anti-cancer drugs have been reported to have many side effects because they kill not only cancer cells but also normal cells. Normal cell death owing to anti-cancer drugs can cause many side effects such as hair loss, tiredness, sore mouth, nausea, vomiting, and appetite loss [6]. However, it has been reported that DIM only kills cancer cells without toxicity to normal cells [24,25], which is a significant advantage of DIM over other anti-cancer drugs because it leads to less complications pertaining to diseases. In addition, the effects of DIM on cancer cells were strong enough to attenuate the proliferation of cancer cells [2]. Therefore, 3,3′-diindolylmethane (DIM) could be a promising therapeutic agent for the prevention and treatment of numerous cancer cells. Indolyl-3-carbinol (I3C) is a bioactive compound found in cruciferous vegetables (Figure 1a). It is rapidly converted to different condensation products, including DIM, in an aqueous and gastric-acidic environment (Figure 1b) [2,26]. DIM inhibits tumor growth via apoptosis in numerous cancer cells [2,27]. DIM has been shown to cause cell cycle arrest in various cancer cells, including esophageal, gastric, and colon cancer cells [27,28,29,30]. Detailed investigations of components of the cell cycle have shown that DIM strongly reduces cyclin-dependent kinase (CDK) 2 activity, which in turn upregulates the expression of p21, a molecule accountable for cell cycle arrest [2]. DIM also initiates reactive oxygen species production and generates stress, triggering DNA damage and ultimately destroying cancer cells. Studies on DIM and cancer cells have proven that DIM triggers cellular endoplasmic reticulum (ER) stress and apoptosis within cancer cells [31]. Preliminary studies have also revealed that the inhibition of autophagy is facilitated by DIM, suggesting that autophagy is the mechanism responsible for cell death [32]. Furthermore, it has been established that DIM exhibits anticancer properties, because it inhibits the growth of human cancer cells by interfering with multiple signaling pathways, restraining invasion, migration, and metastasis and promoting apoptosis [2,23,33,34,35,36,37,38,39,40,41,42,43,44,45,46,47]. Moreover, studies of DIM interaction have shown that DIM can interact with several nuclear transcription factors [48]. Hence, this review is an attempt to summarize the effects of DIM, which acts via multiple cellular and molecular approaches, on various human gastrointestinal cancers. In addition, this review will describe how DIM, with its promising inhibitory activity and potential therapeutic applications for tumorigenesis, may attenuate cancer. It will also highlight current knowledge and perspectives on integrating DIM use into cancer treatment.

## 2. 3,3′-Diindolylmethane and Esophageal Cancer

Globally, esophageal cancer is the eighth most common cancer [49]. Esophageal cancer is classified into esophageal adenomacarcinoma (EAC) and esophageal squamous cell carcinoma (ESCC). EAC occurs in the lower third of the esophagus, whereas ESCC arises in the upper part of esophagus. Socioeconomic status, poor diet, and tobacco use are considered linked to ESCC [50]. Regarding treatment strategies, the options include surgical resection, radiation, and radio-chemotherapy [51]. In addition, early and accurate disease detection is important for longer patient survival. To date, the effects of 3,3′-diindolylmethane (DIM) on EAC have not been reported. Similarly, few studies on the effects of DIM on esophageal cancer have been completed. Investigations performed in our laboratory have suggested that DIM inhibits esophageal cancer proliferation [30]. Our findings have revealed that exposure to 40 μM DIM for 48 h inhibits the growth of ESCC cells by inducing apoptosis and causing G1-phase cell cycle arrest [30]. The population of G1-phase cells increased 48 h after treatment with DIM. In addition, DIM reduces the levels of Cyclin D1 and Cyclin E2 and the activity of cyclin dependent kinase (CDK)4 and CDK6 [30]. The CDK inhibitors p15 and p27 and the apoptotic marker cleaved poly ADP ribose polymerase (PARP) were upregulated, with the activation of caspase-9, in the DIM-treated group [30]. These results suggest that DIM suppresses the development of ESCC via G1-phase cell cycle arrest as well as by inducing apoptosis through the activation of caspase-9.

## 3. 3,3′-Diindolylmethane and Gastric Cancer

Despite extensive research, gastric cancer has one of the highest mortality rates of all cancers worldwide [49]. Studies have shown that vegetable intake is associated with a reduced incidence of gastric cancer [27,52,53]. These effects may be mediated by the inhibition of multiple signaling pathways, reducing cell proliferation, and causing cell cycle arrest, which stimulate apoptotic cell death. A study conducted by our laboratory revealed that 3,3′-diindolylmethane (DIM) suppresses gastric cancer cell growth by activating the Hippo signaling pathway [27]. We found that DIM significantly inhibits gastric cancer cell growth in a dose-dependent manner with G1-phase cell cycle arrest by reducing the levels of the cyclin dependent kinase (CDK) 2, CDK4, CDK6, and Cyclin D1 proteins. DIM also increases p53 levels and pro-apoptotic protein levels [27]. DIM was demonstrated to upregulate core molecules of the Hippo signaling pathway, including phosphorylated (p) large tumor suppressor kinase 1 (LATS1), Mob1, pMob1, p-yes-associated protein (YAP), and ras association domain-containing protein 1 (RASSF1) proteins, while downregulating Yap protein production [27]. DIM increases the binding of RASSF1 to the Mst1/2-LATS1-Mob1 complex, stimulating the Hippo signaling pathway and supporting YAP phosphorylation, which hinders cell proliferation [27]. A similar study by Yin et al. [29] also showed anticarcinogenic effects of DIM. In this study, DIM inhibited gastric cancer cell growth mediated by aryl hydrocarbon receptor (AhR) pathway activation, resulting in G1-phase cell cycle arrest and apoptosis [29]. The overexpression of aryl hydrocarbon is evident during gastric carcinogenesis; thus, modulation of the AhR may contribute to restraining gastric cancer growth [29]. A decline in AhR protein levels and an increase in cytochrome P450 1A1 (CYP1A1) expression in a dose- and time-dependent manner observed after treatment with DIM demonstrate the correlation between AhR and gastric cancer [29]. Another study performed by our laboratory showed that DIM enhances the antitumorigenic properties of paclitaxel through the Akt/forkhaed box protein M1 (FOXM1) signaling pathway in gastric cancer cells [53]. The combination of DIM and paclitaxel highly downregulates the Akt/FOXM1 signaling cascade in gastric cancer cells: FoxM1 and its effector genes, CDK4, p53, and cyclin D1, are also more significantly decreased by combined treatment than by treatment with one compound [53]. In agreement with our results, Ye et al. [52] reported that DIM potentiates paclitaxel-induced antitumor effects and also that DIM potentiates the tumor necrosis factor-related apoptosis-inducing ligand (TRAIL)-induced apoptosis of gastric cancer cells. Evidence indicates that TRAIL selectively kills cancer cells by binding to certain death receptors while not affecting healthy cells [52]. DIM sensitizes gastric cancer cells to the TRAIL-induced inhibition of proliferation and apoptosis by activating protein expression of death receptor 5 (DR5), CCAAT-enhancer-binding protein homologous protein (CHOP), and 78 kDa glucose-regulated protein (GRP78), which may regulate ER stress [52]. Therefore, these findings support the theory that DIM inhibits the growth of human gastric cancer cells. However, in contrast to its antitumor properties, a recent study showed that a low dose of DIM may boost tumorigenesis and stemness in gastric cancer by activating Wnt/β-catenin signaling [54]. Zhu et al. [54] demonstrated both in vitro (wound healing, colony formation assay, and transwell migration) and in vivo (xenograft model) that a low dose of DIM (1–10 μM), as opposed to a high dose, enhances tumorigenicity. These findings provide a different perspective on the safety of DIM for clinical applications and suggest that DIM should be cautiously applied for clinical use to avoid adverse effects on malignancy. Further studies on the effects of low-dose DIM on gastric cancer are needed.

## 4. 3,3′-Diindolylmethane and Colorectal Cancer

Colorectal cancer has the third highest morality rate of all cancers in the US. The incidence of colorectal cancer has dramatically increased in Asian countries, including in South Korea. However, the molecular mechanisms underlying the disease remain unclear. As 3,3′-diindolylmethane (DIM) has been reported to possess cancer chemopreventive properties, the effects of DIM on colorectal cancer have been actively investigated. Gamet-Payrastre et al. [55] first demonstrated the cytotoxic effects of DIM on colon cancer HT29 cells. They reported that low doses of DIM effectively inhibits the cell cycle and reduced the viability of HT29 cells [55]. Bonnesen et al. [56] also demonstrated that DIM stimulates apoptosis and confers protection against DNA damage in human colon adenocarcinoma LS-174 and Caco-2 cells. They found that DIM enhances intracellular defenses against genotoxic agents and prevents colon tumorigenesis by stimulating apoptosis [56]. Despite growing evidence that DIM suppresses colorectal cancer development, the relevant signaling and molecular mechanisms have been not identified. In 2005, Lee et al. [57] attempted to determine the molecular mechanisms by which DIM prevents tumorigenesis in colorectal cancer by examining the TGF-β superfamily gene nonsteroidal anti-inflammatory drug-activated gene-1 (*NAG-1*), which is pro-apoptotic gene. They measured the correlation between *NAG-1* and indole-3-carbinol (I3C) and DIM and its effect on carcinogenesis inhibition. I3C, independent of p53, induces the expression of activating transcription factor 3, which sequentially triggers NAG-1 to suppress cell proliferation in human colorectal cancer (HCT-116) cells. The effect was accentuated when combined with resveratrol [57]. Choi et al. [58] showed that DIM induces G1- and G2/M-phase cell cycle arrest in HT-29 human colon cancer cells. Furthermore, Kim et al. [25] determined the anti-inflammatory effects of DIM on experimental colitis and colon cancer in BALB/c mice. DIM treatment resulted in decreased weight loss, reduced colon shortening, and diminished clinical signs of colitis in mice. These results suggest that DIM has anti-inflammatory and therapeutic qualities against colitis that are associated with colon cancer [25]. Femia et al. also found that DIM combined with curcumin and sulindac reduces colon carcinogenesis in Pirc rats. This study also reported a slight decrease in Survivin-Birc5 expression [56]. Instead of DIM, Fadlalla et al. [59] tested several modified indole compounds on SW480 colon cancer cells. Among these compounds, 3-(2-bromoethyl)-indole (BEI-9) showed the greatest effects on cell viability, wound healing, and cell cycle arrest, according to the results of a NF-κB reporter assay. This led investigators to conclude that the ability of BEI-9 to reduce carcinogenesis should be investigated [59]. Similarly, Lee et al. [60] examined a chemically modified DIM, 1,1-bis(3′-indolyl)-1-(p-substituted phenyl)methane (C-DIM) and its p-hydroxyphenyl analog (DIM-C-pPhOH). C-DIM and DIM-C-pPhOH were found to bind and inactivate nuclear receptor (NR4A1) and also act as a NR4A1 antagonist in lung and pancreatic cancer cells [60]. Lerner et al. [61] examined the expression of N-myc downstream regulated gene-1 (*NDRG1*) in colon cancer HCT-116 cells (well differentiated with a wild-type *p53* gene) and Colo-320 cells (poorly differentiated with a mutant *p53* gene) after treatment with DIM. The results of this study showed increases in NDRG1 expression in poorly differentiated colon cancer cells, causing apoptosis. However, in well-differentiated cells, apoptosis was mediated independently of the NDRG1 pathway [61]. Also, Kim et al. [36] concluded that DIM induces apoptosis through both the intrinsic and extrinsic pathways in colon cancer cells through the activation of caspase 8.

**Figure 2:**
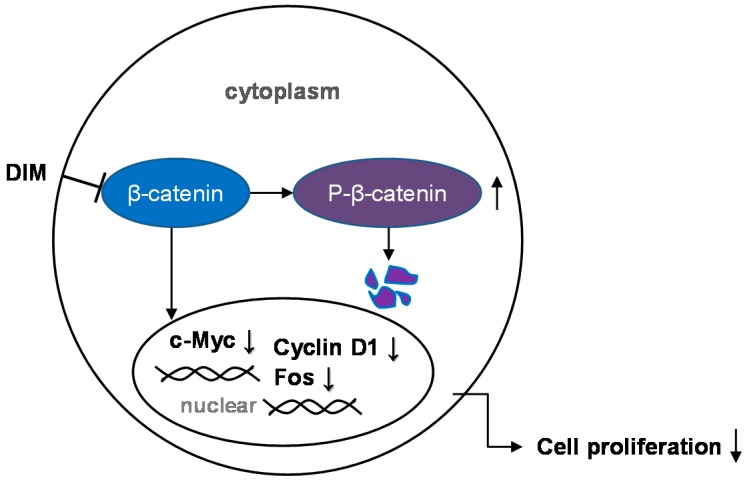
The effect of 3,3′-diindolylmethane on Wnt/β-catenin signaling. The Wnt/β-catenin signaling pathway is modulated by 3,3′-diindolylmethane (DIM). DIM negatively regulates c-Myc, Fos, and Cyclin D1 through the inhibition of β-catenin. Abbreviations: DIM, 3,3′-diindolylmethane; p, phosphorylated; c-Myc, FOS. T arrow: inhibition; Up arrow: increase; down arrow: decrease.

Evidence indicates that impaired regulation of Wnt/β-catenin signaling is one of the causes of colon carcinogenesis and other genetic aberrations. A genome-wide transcriptome analysis performed in our laboratory revealed that DIM alters approximately 1424 genes related to cell proliferation, the cell cycle, and apoptosis. In addition, we found that DIM significantly downregulates β-catenin and c-Myc signaling to inhibit colon cancer cell growth (Figure 2) [62]. Another study conducted by our laboratory showed that DIM significantly represses the migration and invasion of colorectal cancer cells (DLD-1 and HCT-116) [63]. We found that mRNA levels of urokinase type plasminogen activator (uPA) and matrix metalloproteinase (*MMP*)-9 were decreased, and that of E-cadherin were increased. These mRNA levels are mediated by reduced mRNA and protein levels of FOXM1, indicating that DIM inhibits migration and invasion by inactivating FOXM1 [63]. In another study, we found that DIM enhances the toxicity of LY294002, a PI3K inhibitor, in colon cancer cells, as demonstrated by the results of a cell-viability assay, clonogenic assay, and immunoblotting analysis of apoptotic markers [28]. DIM suppresses the proteins involved in the PI3K/Akt pathway (pS473-Akt, pT308-Akt, pPTEN, and pGSK), induces expression of the *RASSF1* gene, and slightly increases *Mst1* and *LATS1* gene expression, thus activating Hippo signaling [28]. In this study, we found that DIM enhances the inhibition of colon cancer proliferation by inhibiting the PI3K/Akt pathway, facilitated by the activation of Hippo signaling [28]. Similarly, another study reported that DIM exhibits a synergistic anticancer activity with capsaicin in human colorectal cancer. These two compounds were also found to synergistically inhibit cell proliferation and induce apoptosis by activating the transcriptional activity of NF-κB and p53. Bhatnagar et al. [24] investigated the combined effect of DIM and butyrate in colon cancer cells containing a mutation in the adenomatous polyposis coli (APC) gene. Butyrate alone does not induce apoptosis in colon cancer cells with the *APC* gene mutation, but combined treatment with DIM accentuates the ability of butyrate to induce apoptosis in these butyrate-resilient cells by downregulating Survivin, both in vitro and in vivo [24]. Our findings, as well as those of several other studies, show the anticancer properties of DIM and demonstrate that a variety of mechanistic approaches may be used in the treatment of colorectal cancer. DIM acts as a suppressive agent in colorectal cancer and influences the proliferation, migration, and invasion of colorectal cancer cells through multiple signaling pathways. In addition, the use of DIM in combination with other compounds is more effective than DIM alone in the treatment of colorectal cancer.

## 5. 3,3′-Diindolylmethane and Liver Cancer

Hepatocellular carcinoma (HCC) is one of the most common causes of cancer mortality worldwide and accounts for an estimated 600,000 deaths annually [49]. HCC is generally prevalent in developing countries in Asia and Africa; however, there has been a recent increase in cases in Europe and the US. [64]. Chronic liver disease, alcoholism, viral hepatitis, and dietary habits are some of the causes of HCC [64,65]. Studies performed in various populations suggest that the consumption of vegetables containing 3,3′-diindolylmethane (DIM), such as cabbage, broccoli, and Brussels sprouts, decreases the risk of developing liver cancer [66,67,68,69]. Numerous studies have shown how DIM works as an anticancer agent through multiple pathways in liver cancer [70,71,72]. As in other cancer cells, DIM inhibits the proliferation, adhesion, migration, and invasion of HCC cells [72,73]. For example, DIM was found to induce apoptosis in SMMC-7721 hepatoma cells [73]. Gong et al. [70] reported that DIM exhibits cytostatic effects in HepG2 cells, inhibits cell cycle progression at the G2/M phase, interacts with DNA in vitro and inhibits DNA synthesis, and blocks mitosis by interfering with mitotic DNA and inhibiting topoisomerases I, IIα, and IIβ, which control the over-winding and under-winding of DNA. Interestingly, Li et al. [72] reported that DIM inhibits the migration, invasion, and metastasis of HCC cells via the phosphorylation of focal adhesion kinase (FAK, tyr397) with decreased expression of MMP-2 and MMP-9, because FAK and MMP2/9 are upregulated in liver cancer cells (SMC-7721 and MNCC-97H) and are responsible for malignancy [72]. In this study, Li et al. [72] demonstrated that oral administration of DIM inhibits hepatic tumor nodules and lung-metastatic nodules compared with a control group of BALB/c nude mice. In addition, Paltsev et al. [74] conducted a preclinical trial to evaluate the pharmacokinetics and bioavailability of DIM in animal models. Their data showed that a novel pharmacologic DIM substance with high bioavailability may be a promising targeted chemopreventive agent, indicating that DIM could be used to prevent the progression of liver cancer [74].

In contrast, some studies have shown DIM to be a promoter of liver tumorigenesis. Data obtained by Tilton et al. [75], using vitellogenin (VTG) and cytochrome P450 1A1 (CYP1A1) as markers for the activation of the estrogen receptor and AhR in fish and other animal models, suggested that indole-3-carbinol (I3C) enhances hepatocarcinogenesis through the estrogen signaling pathway, especially at lower nutritional levels. In this study, the roles of I3C and DIM were found to be similar to 17β-estradiol (E2) at the protein level in data obtained from a correlation analysis of gene expression profiles [75]. Further studies have confirmed that the response of VTG to DIM in trout is similar to that of E2, which is an estrogen receptor agonist in rainbow trout [76]. Moreover, I3C has been shown to have unsolicited tumor-enhancing activities in the rat liver [77]. In this study, Parkin and Malejka-Giganti [77] found that I3C promotes tumor activities, whereas DIM appeared to suppress tumor growth in mammary tumor-bearing rats. Therefore, the results of studies on the effects of I3C have been contradictory. Further studies in humans are needed to determine the long-term effects of I3C and DIM on liver cancer.

## 6. 3,3′-Diindolylmethane and Pancreatic Cancer

Pancreatic cancer is the fourth leading cause of death attributable to cancer in the US, and its OS rate is small [78,79]; thus, it is vital to understand the molecular mechanisms involved. The clinical management of pancreatic cancer is a complex challenge because of resistance to conventional therapeutics [78,79]. Evidence from some studies has indicated that 3,3′-diindolylmethane (DIM) inhibits the proliferation of Panc-1 and Panc-28 pancreatic cancer cells by inducing apoptosis through the upregulation of DR5. DR5 is a death receptor associated with ER stress and the cleavage of caspase 8, caspase 3, Bid, and PARP [80]. Intriguingly, a study performed by Hong et al. [81] investigated the link between 1,1-bis(3’-indolyl)-1-(p-trifluoromethylphenyl)methane (DIM-C-pPhCF3) and peroxisome proliferator-activated receptor γ (PPARγ) in panc-28 pancreatic cancer cells. Using fluorescence-activated cell sorting analysis, they found that DIM-CpPhCF3 is more effective at inhibiting G0/G1–S phase progression. These results are associated with decreased phosphorylation of the retinoblastoma protein and increased expression of p21 protein and mRNA. DIM-C-pPhCF3 was found to induce p21 expression through a new pathway that includes interactions between peroxisome proliferator-activated receptor gamma (PPARγ) and both specificity protein (Sp)1 and Sp4 proteins bound to the proximal GC-rich motifs of the p21 promoter [81]. In another study on pancreatic cancer, Banerjee et al. [82] found that the use of DIM enhances the chemosensitivity of several chemotherapeutic agents (cisplatin, gemcitabine, and oxaliplatin) compared with monotherapy. Similar effects were observed in a study by Ali et al. [83], which reported significant decreases in cell viability, the onset of apoptosis, decreases in the phosphorylation of epidermal growth factor receptor (EGFR), and NF-κB DNA binding activity in MiaPaCa cells after treatment with DIM in combination with erlotinib. In this experiment, which used an orthotopic model of pancreatic cancer, DIM potentiated the apoptosis-inducing effect of erlotinib in vitro and in vivo [83]. The fact that these in vitro characteristics were also evident in an in vivo environment provides stronger evidence for the use of DIM in patients with pancreatic cancer exhibiting overexpression of EGFR and NF-κB [83]. In addition, in subsequent clinical trials, the authors showed that the simultaneous inhibition of cyclooxygenase-2, EGFR, and NF-κB by DIM and erlotinib or gentamycin results in significant decreases in cell viability [84]. These results imply that patients with tumors that show high levels of EGFR, cyclooxygenase-2, and NF-κB can be treated with combinatorial therapies [84]. Also, DIM pretreatment increased the apoptosis even at lower doses of chemotherapeutic drugs by downregulation of NF-κB and its related genes such as Bcl-xL, X-linked inhibitor of apoptosis protein (XIAP), cIAP, and survivin [82]. Azmi et al. studied the chemoprevention of pancreatic cancer cells by DIM in relation to prostate apoptosis response-4 (*Par-4*) gene, which induces apoptosis in prostate cancer cells. Their results indicated that low doses of B-DIM induce Par-4 b expression and result in apoptotic death when combined with other chemotherapeutic drugs.

miR-221 is highly upregulated in pancreatic cancer cells and tumor tissues and that patients with pancreatic cancer that exhibits higher expression of miR-221 have shorter lifespans. This suggests that miR-221 is an oncogenic microRNA (miRNA) [85]. Sarkar et al. [85] found that treatment with DIM downregulates miR-221 expression and upregulates phosphatase and tensin homolog (PTEN), p27, p57, and p53 upregulated modulator of apoptosis (PUMA) expression, thus inhibiting the proliferation and migration of MiaPaCa-2 and Panc-1 cells. Li et al. [86] also investigated the connection between miR-146a and pancreatic carcinogenesis in Colo357 and Panc-1 pancreatic cancer cells. They reported lower expression of miR-146 in pancreatic cancer cells than in normal human pancreatic duct epithelial cells and found that re-expression of miR-146a subdued malignancy by downregulating EGFR, NF-κB, IRAK-1, interleukin-1 receptor-associated kinase 1 (IκBα), and metastasis associated 1 family member 2 (MTA-2) [86]. Their results prove that B-DIM increases miR-146a and hinders invasion through decreases in EGFR and NF-κB [86]. A similar study conducted by Li et al. [87] suggested that epithelial–mesenchymal transition (EMT) is controlled by the level of miRNA expression and is responsible for the biology of tumor progression. The investigators compared miRNA expression between gemcitabine-sensitive and -resistant pancreatic cancer cells and examined the effect of DIM on miRNAs. They found that the expression of miR-200 and let-7 is considerably decreased in gemcitabine-resistant cells with EMT characteristics [87]. When miRNA-200 was re-expressed by transfection in gemcitabine-resistant cells or the cells were exposed to DIM, their EMT morphology reverted to the epithelial phenotype, with the downregulation. These results suggest that DIM functions as a miRNA regulator and can reverse the EMT phenotype [87]. Taken together, these results demonstrated the suppressor properties of DIM and the potential use of DIM as an inhibiting agent in pancreatic cancer. Altogether, this review article offers a comprehensive outline of biological mechanisms, targets, and modes of action of DIM in gastrointestinal cancer (Table 1), signifying that DIM could prospectively be beneficial for chemoprevention and as a cancer therapeutic. DIM could be useful as an adjunct to conventional therapeutics for new perspectives on prevention and treatment of gastrointestinal cancers in the future.

Despite the fact that DIM exhibits a great safety and efficacy profile, because of its unknown adverse effects and low bioavailability, it remains challenging to employ it in clinical practice. For instance, a recent study by Zhu et al. [54] showed that a low dose of DIM stimulated gastric cancer tumorigenesis which indicated a different perspective of DIM’s safety. Therefore, further extensive laboratory investigation with emerging different technologies, including the use of animal models of human diseases, computational analysis, and molecular techniques, are necessary to investigate novel signaling pathways involved in the action of DIM in gastrointestinal cancers. Moreover, translational and clinical trials in the advancement of our knowledge are required to verify whether DIM could satisfy its potential as a chemopreventive/therapeutic agent against human gastrointestinal cancers.

## 7. Conclusions

**Figure 3:**
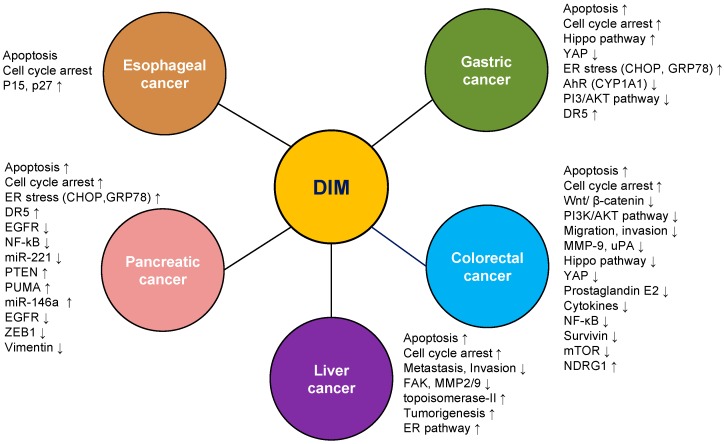
Schematic representation of the effect of 3,3′-diindolylmethane on gastrointestinal cancer. 3,3′-diindolylmethane regulates several molecular interactions and signaling pathways to attenuate esophageal, gastric, colorectal, liver, and pancreatic cancer. Abbreviations: DIM, 3,3′-diindolylmethane. Up arrow: increase; down arrow: decrease.

The studies to date have showed in vitro and in vivo that DIM exhibits both anticancer and tumor-promoting properties in gastrointestinal cancer cells via several signaling pathways (Figure 3 and Table 1). Such studies increase our understanding of the biological mechanisms underlying gastrointestinal cancer and reveal potential targets for the treatment of the condition. The influence of DIM on processes including apoptosis, cell-cycle arrest, invasion, metastasis, and cell signaling should be taken into account and further studied using more sophisticated technologies with in vivo and in vitro studies. Understanding how DIM effects on cancer cells may eventually lead to initial new avenues for therapeutic intervention for gastrointestinal cancer treatment.

## Figures and Tables

**Table 1:** Summary of the action of 3,3′-diindolylmethane (DIM) on gastrointestinal cancers and its associated functions.

Cancer Type	Model Used (Cell Line/Animal)	Mechanism of Action
Esophageal cancer	TT, TE-8, TE-12	Cell cycle arrest at G1 phase [30]
Gastric cancer	SNU-1, SNU-484	Inhibition of cell growth by activation of hippo signaling pathway [27]
	SNU638	Inhibition of cell growth by downregulation of Akt/FoxM1 signaling pathway [53]
	BGC-823, SGC-7901	Activation of TRAIL induced apoptosis [52]
	HGC-27, SGC-7901, MGC-803	Induction of carcinogenesis by activation of Wnt4 signaling [54]
Colorectal cancer	HT29, Caco-2	Reduction of cell viability by cell cycle arrest [55]
	LS-174, Caco-2	Induction of apoptosis [56]
	HCT-116	Inhibition of cell growth by triggering NAG-1 [57]
	HT-29	Cell cycle arrest at G1 and G2/M phase [58]
	BALB/c mice	Induction of anti-inflammatory effect [25]
	SW480	Activation of NF-κB [59]
	RKO, SW480	Inhibition of cell growth by antagonizing NR4A1 [60]
	HCT-116, Colo-320	Inhibition of cell growth by apoptosis mediated by NDRG1 [61]
	HCT116, HT-29	Induction of apoptosis by activation of caspase-8 [36]
	DLD-1, HCT116	Inhibition of cell growth by inactivation of β-catenin/c-Myc [62]
	DLD-1, HCT-116	Inhibition of cell growth by inactivation of FOXM1 [63]
	HCT116	Inhibition of cell growth by activation of Hippo signaling pathway [28]
	HT-29	Inhibition of cell growth by downregulation of survivin [24]
Liver cancer	SMMC-7721	Induction of apoptosis [73]
	HepG2	Cell cycle arrest at G2/M phase [70]
	SMMC-7721, MNCC-97H	Inhibition of cell proliferation via phosphorylation of FAK [72]
	Rainbow trout (fish)	Induction of tumorigenesis via estrogen signaling pathway [75,76]
Pancreatic cancer	Panc-1, Panc-28	Induction of apoptosis by upregulation of DR-5 [80]
	Panc-28	Inhibition of cell growth through upregulation of p21 protein [81]
	Panc-28, COLO-357, Panc-28	Augmentation of apoptosis by downregulation of NF-κB [82]
	MiaPaCa	Augmentation of apoptosis when combined with erlotinib [83]
	MiaPaCa-2, Panc-1	Inhibition of cell growth by downregulation of miR-221 [85]
	Colo357, Panc-1	Inhibition of cell growth via upregulation of miR-146 [86]
	MiaPaCa-2, Panc-1, Aspc-1	Reversal of EMT by upregulation of miR-200 [87]
